# Bilateral Optic Neuritis in Paediatric Acute Disseminated Encephalomyelitis: A Case Report of Clinical Challenges and Management Outcomes

**DOI:** 10.7759/cureus.106441

**Published:** 2026-04-04

**Authors:** Nur Izzah Husna Saaid Zaidun, Khairul Anwar Ibrahim, Khairidzan Mohd Kamal

**Affiliations:** 1 Ophthalmology, International Islamic University Malaysia (IIUM), Kuantan, MYS

**Keywords:** acute disseminated encephalomyelitis, demyelinating disease, optic neuritis, paediatric optic neuritis, retrobulbar optic neuritis

## Abstract

Paediatric optic neuritis is a rare inflammatory demyelinating condition that frequently presents with bilateral involvement and a generally favourable prognosis compared to adult-onset forms. We report the case of a seven-year-old boy with a history of thalassemia trait and mild autism who presented with a one-day history of sudden bilateral painful vision fluctuation, headache, and fever. Initial visual acuity (VA) was 6/12 in the right eye and 6/30 in the left eye, with normal optic nerve function test and fundoscopic examination indicating retrobulbar involvement. While initial computed tomography (CT) imaging was unremarkable, urgent magnetic resonance imaging (MRI) of the brain and orbit revealed features consistent with inflammatory demyelinating disease. Extensive investigations, including cerebrospinal fluid (CSF) analysis and testing for myelin oligodendrocyte glycoprotein (MOG-IgG) and aquaporin-4 (AQP4-IgG) antibodies, were negative, suggesting a seronegative monophasic demyelinating event such as acute disseminated encephalomyelitis (ADEM) spectrum. The patient was treated with high-dose intravenous methylprednisolone (IVMP) followed by a six-week oral steroid taper, resulting in a rapid recovery of VA to 6/6 bilaterally. At the six-month follow-up, the patient remained asymptomatic with stable neuroimaging. This case highlights the critical role of early contrast-enhanced MRI in diagnosing paediatric retrobulbar optic neuritis and the effectiveness of prompt, aggressive corticosteroid therapy in achieving excellent visual outcomes in seronegative paediatric demyelinating disorders.

## Introduction

Optic neuritis is an inflammatory condition of the optic nerve that can lead to significant visual impairment [1]. It represents a frequent clinical manifestation of demyelinating disorders within the paediatric population [2]. While historically considered a rare condition, affecting approximately 0.15 to 0.57 per 100,000 children [3], recent epidemiological data from 2023 indicate a significant upward trend in prevalence [4]. The paediatric clinical presentation is distinct from that seen in adults, often being characterized by more severe initial visual loss yet maintaining a generally favourable prognosis for recovery. Common clinical features include acute vision loss (either unilateral or bilateral), periocular pain, dyschromatopsia, and optic disc swelling [1].

Accurate differentiation of the underlying aetiology is crucial, as it determines long-term management and prognosis. The spectrum of paediatric optic neuritis encompasses both infectious and non-infectious causes, with the most common being demyelinating diseases, namely, neuromyelitis optica spectrum disorder (NMOSD), myelin oligodendrocyte glycoprotein antibody-associated disease (MOGAD), multiple sclerosis (MS), and Leber’s hereditary optic neuropathy (LHON) [5]. While acute disseminated encephalomyelitis (ADEM) remains a relatively uncommon condition, with an estimated annual incidence of 0.07 to 0.9 per 100,000 children [6], it is characterized as an acute, multifocal demyelinating disorder involving the brain (encephalopathy) and spinal cord. Involvement of the optic nerve (optic neuritis) is less frequent in ADEM and typically follows a preceding infectious prodrome or, more rarely, vaccination [7].

This case report aims to demonstrate the challenges in managing bilateral retrobulbar optic neuritis in a paediatric patient secondary to ADEM.

## Case presentation

A seven-year-old boy with a known thalassemia trait and mild autism presented to the Sultan Ahmad Shah Medical Centre with a one-day history of sudden-onset bilateral painful fluctuating vision. This was associated with headache and fever; however, there was no history of nausea or vomiting.

Upon examination, visual acuity (VA) using Lea symbols was 6/12 in the right eye (RE) and 6/30 in the left eye (LE). Intraocular pressures were 11 mmHg bilaterally (BE). Optic nerve function tests, including light reflex and colour vision, were within normal limits, and the patient was orthophoric. Formal perimetry was attempted but was unreliable due to limited patient cooperation. Anterior segment examination was unremarkable, and dilated fundoscopy revealed no optic disc swelling in either eye. A systemic neurological examination was also unremarkable.

**Figure 1:**
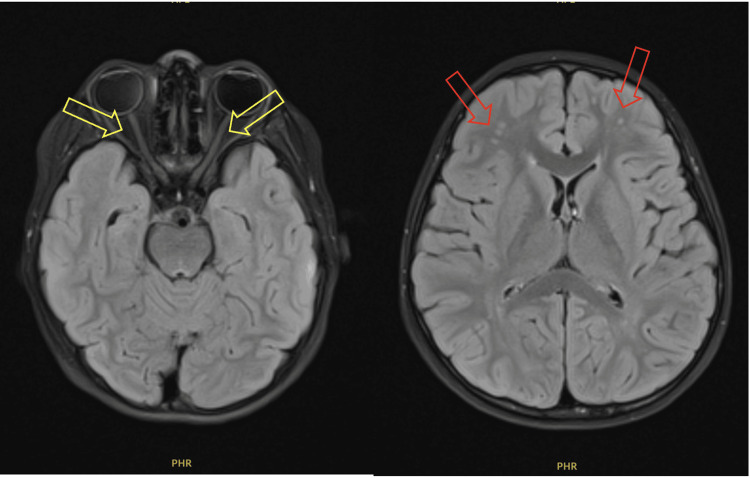
Axial cut of MRI brain and orbit T2-weight post gadolinium at initial presentation showing normal optic nerve (left photo) (yellow arrow) and multiple small hyperintense lesion at subcortical and juxtacortical region to suggest inflammatory demyelinating disease (right photo) (red arrow).

An urgent non-contrast computed tomography (CT) of the brain showed no acute intracranial pathology or space-occupying lesions. Over the following days, the patient’s vision continued to fluctuate. Given the inconclusive clinical findings, urgent magnetic resonance imaging (MRI) of the brain and orbits was performed. The MRI revealed features of an inflammatory demyelinating disease involving the optic nerves, consistent with the patient's presentation (Figure 1).

The patient was co-managed with the paediatric neurology team from the fifth day of illness. Hematological and biochemical investigations were within normal limits and did not suggest an active infection (Table 1). A lumbar puncture was performed to confirm the diagnosis and rule out infectious aetiologies. The patient was started on high-dose intravenous methylprednisolone (IVMP) at 30 mg/kg/day (500 mg twice daily) and IV ceftriaxone (50 mg/kg twice daily) to cover for potential meningoencephalitis.

**Table 1:** Summary of investigation for the patient WBC: white blood cell, HBP: hemoglobin, CSF: cerebrospinal fluid, MOG: myelin oligodendrocyte glycoprotein, PCR: polymerase chain reaction

Laboratory investigations	Results	Unit	Reference range
WBC	9.7	10^9/L	4.08-11.37
HB	109	g/L	116.0-151.0
Platelet	487	10^9/L	171-399
CRP	0.07	mg/dL	<1.0
Blood culture sensitivity	No growth		
CSF analysis			
Appearance	Clear		
Glucose	3.8	mmol/l	2.2-3.9
Protein	0.31	g/L	0.15-0.45
Albumin	162.4	mg/L	100-300
Culture	Negative		
Gram stain	No organism seen		
India ink	Capsulated yeast not seen		
Aquaporin 4	Negative		
MOG	Negative		
Meningitis PCR panel	Not detected		

**Figure 2:**
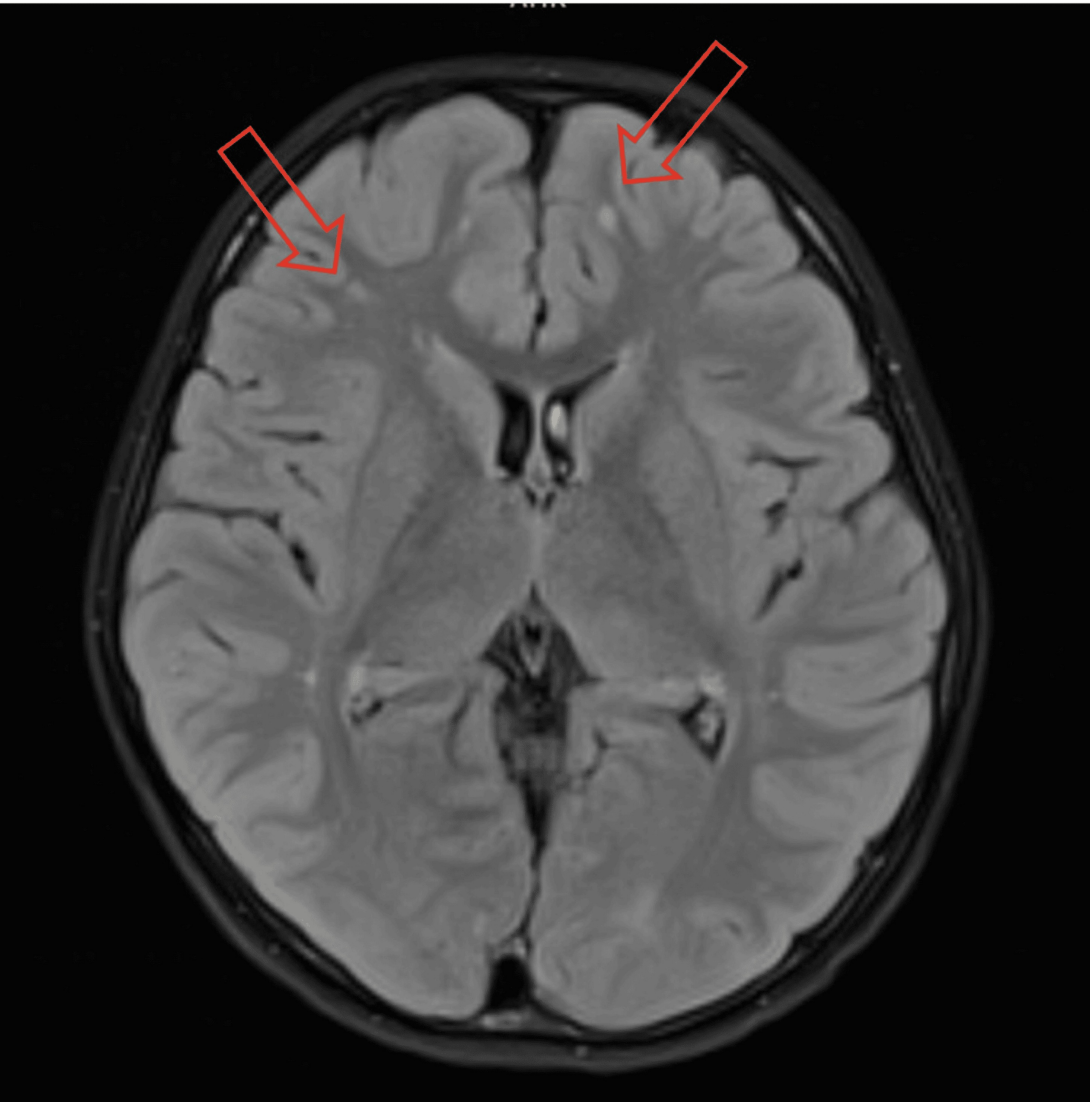
Repeated MRI brain and orbit at six-month follow up showing no changes of lesion at cortical region as compared to the previous MRI which indicates stable demyelinating lesion.

Following the initiation of IVMP, the patient’s vision rapidly improved to 6/6 in both eyes. Subsequent cerebrospinal fluid (CSF) analysis was negative for bacterial and viral cultures, and serological testing for AQP4-IgG and MOG-IgG was negative (Table 1). The patient completed a five-day course of IVMP and was discharged on a six-week tapering dose of oral prednisolone. At the six-month follow-up, VA remained stable at 6/6 BE, with no recurrence of headaches or visual fluctuations. A repeat MRI showed stable demyelinating lesions with no new activity (Figure 2).

## Discussion

Paediatric optic neuritis is a distinct clinical entity, characterized by unique epidemiological trends and manifestations that differ significantly from adult-onset disease. In this case, the seven-year-old patient presented with acute bilateral vision loss, a hallmark feature of optic neuritis in younger children. The presentation was consistent with retrobulbar optic neuritis, as evidenced by the inflammatory findings on MRI despite a normal funduscopic appearance. Literature indicates that children under the age of 10 are significantly more likely to present with bilateral involvement, occurring in up to 72% of cases, whereas unilateral involvement is more prevalent in adolescents and adults [1]. Interestingly, a regional study on pediatric optic neuritis in Malaysia reported a female predominance and a high incidence of visible optic disc swelling (papillitis) [3,8]. Our case differs by presenting as retrobulbar neuritis in a male patient, highlighting the clinical heterogeneity within the paediatric population. The absence of disc swelling in a seven-year-old, as seen in this case, is atypical and requires urgent neuroimaging, as the lack of funduscopic findings can lead to a misdiagnosis of non-organic vision loss or malingering, especially in a child with comorbid autism [3].

The specific serology findings for this patient highlighted the complexity of seronegative paediatric demyelinating disorders. While current diagnostics emphasized the roles of Myelin Oligodendrocyte Glycoprotein (MOG-IgG) and Aquaporin-4 (AQP4-IgG) antibodies, some patients may remain seronegative, which poses a significant challenge for definitive diagnosis [9]. In this case, the patient’s systemic symptoms together with the MRI findings of inflammatory demyelinating disease suggested a possible link to ADEM. Although the patient did not manifest encephalopathy, which is the clinical hallmark of ADEM, bilateral optic neuritis itself is frequently the presenting feature of a monophasic demyelinating event for the ADEM spectrum [10]. The exclusion of infective causes through negative cerebrospinal fluid (CSF) cultures and viral parameters in the present case further supports an immune-mediated aetiology, reinforcing the need to relate clinical, serological, and neuroimaging data.

Neuroimaging is crucial in the management of paediatric patients with suspected retrobulbar neuritis, particularly when funduscopic examination is unremarkable. In this case, early MRI of the brain and orbit plays an important role in identifying inflammatory lesions that could not be localized by clinical examination. Advanced imaging not only confirms the diagnosis by demonstrating gadolinium enhancement or T2-weighted signal abnormalities within the optic nerves but also aids in identifying silent white matter lesions that may indicate a higher risk for future demyelinating events [2]. The stable six-month follow-up MRI in our patient shows no new silent lesions, which further supports a monophasic ADEM-spectrum event rather than the early stages of a chronic relapsing disorder like MS or NMOSD.

The management protocol for this patient followed the established standard of high-dose IVMP followed by a gradual tapering of oral steroids. This aggressive approach is designed to hasten the visual recovery and alleviate the inflammatory cascade. The favourable prognosis observed in this case, with VA returning to 6/6 bilaterally, aligns with recent longitudinal studies indicating that over 80% of paediatric patients achieve a best-corrected VA of 20/40 or better within six months of treatment [11]. In our case report, despite the excellent initial recovery and stable six-month follow-up findings, long-term monitoring remains necessary for this patient as seronegative bilateral optic neuritis in childhood carries a risk of relapse [12]. Continuous follow-up is crucial to detect early signs of relapse or progression. Timely introduction of disease-modifying therapies, when indicated, ensures the long-term preservation of visual and neurological function during childhood development [5].

## Conclusions

Paediatric optic neuritis remains a critical clinical entity that requires immediate multidisciplinary coordination between ophthalmology, paediatrics, and neurology teams to prevent permanent visual morbidity. This case of a seven-year-old boy with bilateral retrobulbar optic neuritis illustrates the unique diagnostic challenges of paediatric demyelinating disorders, particularly when optic disc swelling is absent. The negative serology for MOG-IgG and AQP4-IgG antibodies and systemic symptoms suggests a seronegative, presumptive ADEM-spectrum event. Hence, early contrast-enhanced MRI was critical in identifying inflammatory lesions that were clinically unremarkable on funduscopic examination, facilitating timely and directed intervention.

The rapid restoration of 6/6 VA following high-dose IVMP suggests that prompt and aggressive corticosteroid therapy can be effective in achieving favourable outcomes in similar paediatric cohorts. However, given the observational nature of this single case and the potential for seronegative presentations to evolve into relapsing conditions such as multiple sclerosis or NMOSD, longitudinal clinical and radiological monitoring is required. This approach ensures the early detection of any future demyelinating events and the long-term preservation of neurological function.
